# A97 DEVELOPMENT OF PREDICTION MODELS FOR THE TRIAGING OF REFERRALS OF INDIVIDUALS WITH SUSPECTED INFLAMMATORY BOWEL DISEASE TO IMPROVE PROMPT ACCESS TO CARE

**DOI:** 10.1093/jcag/gwab049.096

**Published:** 2022-02-21

**Authors:** J St-Pierre, A Frolkis, C Seow, J Oshiomogho, G Bindra, J Heatherington, G G Kaplan, R Panaccione, K L Novak, Y Nasser, H Jijon

**Affiliations:** 1 Medicine, University of Calgary, Calgary, AB, Canada; 2 University of Calgary, Calgary, AB, Canada; 4 Medicine and Community Health Sciences, University of Calgary, Calgary, AB, Canada; 6 Gastroenterology, University of Calgary, Calgary AB, AB, Canada; 7 Division of Gastroenterology and Hepatology, Department of Medicine, University of Calgary, Calgary, AB, Canada

## Abstract

**Background:**

The negative impact of a delayed inflammatory bowel disease (IBD) diagnosis has been well established. We created a clinical pathway referred to as the “High-Risk IBD clinic” within a centralized referral program in a tertiary referral centre, in order to improve access to subspecialist care for individuals suspected but not yet diagnosed with IBD. Despite the creation of this specialized clinic, wait times continue to be above the recommended benchmarks established by the Canadian Association of Gastroenterology (CAG).

**Aims:**

The purpose of our study was to create predictive models to identify factors associated with an IBD diagnosis in order to improve triage of referrals of individuals with features highly suggestive of IBD. We hypothesized that features suggestive of IBD could be used to create discriminating prediction models between IBD and IBS.

**Methods:**

We conducted a retrospective cohort study of referrals to the High-Risk IBD clinic from February 2014 to December 2018. Referral information, investigations, endoscopic findings and final diagnosis were obtained from 316 consented individuals. Information required included symptoms (e.g. diarrhea, abdominal pain, rectal bleeding), risk factors (e.g. family history, rheumatological disease) and investigations (e.g. hemoglobin, CRP, abdominal imaging). Univariate logistic regression was performed to explore the association between factors included in the referral form, and a diagnosis of Crohn’s disease (CD) and ulcerative colitis (UC). For creation of predictive models, any variable with a p-value of <0.1 in univariate logistic regression was selected for entry into the multivariate model for CD and UC.

**Results:**

For UC, the predictive model included weight loss, the presence of rectal bleeding and abdominal pain. Using these criteria, the sensitivity and specificity of the model were 62.5% and 74.1%, respectively. The negative predictive value (NPV) was high at 94.2%. For CD, the predictive model included male gender, elevated CRP, presence of anemia and presence of weight loss. The sensitivity and specificity of this model were 61.7% and 71.2%, respectively. As for UC, the NPV was also high (89.2%). For IBS, the most common diagnosis encountered in patients referred to the HR-IBD clinic, the model included absence of weight loss, presence of abdominal pain and female gender. The sensitivity and specificity were 71.6% and 64.0%, respectively. The positive predictive value was 60.6% and NPV was 74.5%.

**Conclusions:**

We established predictive tools associated with a final diagnosis of IBD and IBS as a means to expedite the care of individuals with undiagnosed IBD.

**Funding Agencies:**

CCC

